# Feasibility of sentinel node navigated surgery in high-risk T1b esophageal adenocarcinoma patients using a hybrid tracer of technetium-99 m and indocyanine green

**DOI:** 10.1007/s00464-021-08551-6

**Published:** 2021-05-27

**Authors:** Anouk Overwater, Bas L. A. M. Weusten, Jelle P. Ruurda, Richard van Hillegersberg, Roel J. Bennink, Bart de Keizer, Sybren L. Meijer, Lodewijk A. A. Brosens, Roos E. Pouw, Jacques J. G. H. M. Bergman, Mark I. van Berge Henegouwen, Suzanne S. Gisbertz

**Affiliations:** 1 Department of Gastroenterology and Hepatology, St. Antonius Hospital, Koekoekslaan 1, 3435 CM Nieuwegein, The Netherlands; 2 Department of Gastroenterology and Hepatology, University Medical Center Utrecht, Utrecht University, Utrecht, The Netherlands; 3 Department of Surgery, University Medical Center Utrecht, Utrecht University, Utrecht, The Netherlands; 4 Department of Radiology and Nuclear Medicine, Amsterdam University Medical Centers, Amsterdam, the Netherlands; 5 Department of Radiology and Nuclear Medicine, University Medical Center Utrecht, Utrecht University, Utrecht, the Netherlands; 6 Department of Pathology, Amsterdam University Medical Centers, Amsterdam, the Netherlands; 7 Department of Pathology, University Medical Center Utrecht, Utrecht University, Utrecht, the Netherlands; 8 Department of Gastroenterology and Hepatology, Amsterdam University Medical Centers, Amsterdam, the Netherlands; 9 Department of Surgery, Cancer Center Amsterdam, Amsterdam UMC, University of Amsterdam, Amsterdam, the Netherlands

**Keywords:** Sentinel lymph node, Lymph node excision, Esophageal neoplasms, Esophageal adenocarcinoma, Indocyanine green

## Abstract

**Background:**

Minimally invasive esophagectomy with two-field lymphadenectomy is standard of care for T1b esophageal adenocarcinoma (EAC) with a high risk of lymph node metastasis. Sentinel node navigation surgery (SNNS) is a well-known concept to tailor the extent of lymphadenectomy. The aim of this study was to evaluate the feasibility and safety of SNNS with a hybrid tracer (technetium-99 m/indocyanine green/nanocolloid) for patients with high-risk T1b EAC.

**Methods:**

In this prospective, multicenter pilot study, 5 patients with high-risk T1b EAC were included. The tracer was injected endoscopically around the endoscopic resection scar the day before surgery, followed by preoperative imaging (lymphoscintigraphy/SPECT-CT). During surgery, first the SNs were localized and resected based on preoperative imaging and intraoperative gammaprobe- and fluorescence-based detection, followed by esophagectomy. Primary endpoints were the percentage of patients with detectable SNs, concordance between preoperative and intraoperative SN detection, and the additive value of indocyanine green.

**Results:**

SNs could be identified and resected in all patients (median 3 SNs per patient, range 2–7). There was a high concordance between preoperative and intraoperative SN detection. In 2 patients additional peritumoral SNs were identified with fluorescence-based detection. None of the resected lymph nodes showed signs of (micro)metastases and no nodal metastases were detected in the surgical resection specimen.

**Conclusions:**

SNNS using technetium-99 m/indocyanine green/nanocolloid seems feasible and safe in patients with high-risk T1b EAC. Indocyanine green fluorescence seems to be of additive value for detection of peritumoral SNs. Whether this approach can optimize selection for esophagectomy needs to be studied in future research.

**Supplementary Information:**

The online version contains supplementary material available at 10.1007/s00464-021-08551-6.

## Introduction

Early esophageal adenocarcinoma (EAC) with invasion limited to the (sub)mucosa can be resected endoscopically. For mucosal (T1a) EAC, endoscopic resection is considered curative treatment, because the risk of lymph node metastases (LNM) for these tumors is negligible [1]. Submucosal (T1b) EAC can be divided into two risk categories based on histopathological characteristics of the endoscopic resection specimen. If only histopathological features with a low risk of LNM are present (i.e., tumor free deep resection margins, submucosal infiltration < 500 µm, good/moderate tumor differentiation, and no lymphovascular invasion), endoscopic resection is still considered curative, since for these lesions lymphatic spread of tumor cells to adjacent lymph nodes is highly exceptional (< 2%) [2, 3]. If one or more of the histopathological features with a high risk of LNM is present (i.e. tumor positive deep resection margins, submucosal invasion > 500 µm, poor tumor differentiation, and/or lymphovascular invasion), the risk of concomitant LNM is higher (0–37%) [2–5], and current clinical guidelines recommend esophagectomy [6, 7].

However, esophagectomy is an invasive procedure associated with significant morbidity (up to 56%), mortality (up to 4.6%) and reduced postoperative quality of life [8, 9]. Since T1b EAC can often be removed completely with endoscopic resection, additional surgery is only performed because of potential LNM. Yet, with LNM rates between 0 and 37% [2–5], surgical resection of the esophagus and all locoregional lymph nodes is unnecessary in the majority of patients.

Ideally, if a radical endoscopic resection of a high-risk T1b EAC has been performed, additional treatment would be tailored based on lymph node involvement. Sentinel node navigated surgery (SNNS) is a concept already extensively applied to personalize and limit lymph node dissection for other malignancies, and has also previously been applied for EAC [10]. Especially T1 EAC is associated with good results, while patients with more advanced carcinomas are being considered non-suitable candidates because of lymph vessel destruction by the tumor and neoadjuvant therapy [11]. The majority of T1b EAC patients will have no tumor cells in the sentinel node (SN) and thus no need for additional treatment. This alternative approach would be less invasive, leave the upper gastrointestinal anatomy intact with function preservation of the esophagus and stomach and thereby possibly lead to lower morbidity and mortality and better postoperative quality of life compared to esophagectomy.

In a preceding study, we found SNNS using technetium-99 m(^99m^Tc)-nanocolloid to be feasible and safe in patients with high-risk T1b EAC. However, in one patient one peritumoral tumor positive SN could not be identified due to high tracer activity at the injection site, known as the shine through effect [12]. Indocyanine green (ICG), a green dye, can be visualized with near-infrared (NIR) light during surgery and enhances intraoperative visualization of peritumoral lymph nodes [13]. Combining these radioactive (^99m^Tc) and fluorescence (ICG) techniques has shown promising results for SN mapping in other malignancies, but has not been evaluated in EAC [14–16].

This is the first study to evaluate the feasibility, accuracy and safety of SNNS using a *hybrid* tracer of ICG and ^99m^Tc-labelled nanocolloid in patients with high-risk T1b EAC. We hypothesize that this is feasible and safe, and that the addition of ICG improves peritumoral SN identification. Ultimately, the goal is to use SNNS to specifically select patients that will benefit from additional treatment after radical endoscopic resection of high-risk T1b EAC.

## Material and methods

### Study design and patient population

For this pilot study, patients were included in three expert centers for the treatment of Barrett-related neoplasia in the Netherlands (St. Antonius Hospital, Nieuwegein; Amsterdam UMC, location AMC, Amsterdam; UMC Utrecht, Utrecht). Patients were eligible when diagnosed with high-risk T1b EAC based on histopathological evaluation of the endoscopic resection specimen, with no clinical signs of lymph node involvement as determined by preoperative staging, and planned for additional esophagectomy. Since all study participants were planned for additional surgery, an incomplete endoscopic resection was not an exclusion criterion. Neo-adjuvant (chemo) radiation therapy, another primary tumor, known allergy for ^99m^Tc-nanocolloid or ICG, previous surgery or comorbidity interfering with the procedures, were exclusion criteria.

### Sentinel node navigation surgery

Patients were admitted to the hospital one day before surgery. Submucosal endoscopic injection of 2 cc of a *combined* ^99m^Tc-ICG-nanocolloid tracer (100 MBq, 0.17 mg ICG; GE Healthcare, Chicago, Illinois, USA) was performed divided over four quadrants around the endoscopic resection scar. The combination with nanocolloid increases the hydrodynamic diameter of the tracer and ensures that the tracer is retained in the SNs and remains visible during surgery the next day [10, 17]. Subsequently, planar images were made using a gamma camera 15 and 120 min after injection of the tracer, the latter directly followed by SPECT/CT. Pre-operative imaging comprised the area extending from the neck to the upper abdomen to ensure identification of the anatomical location of all SNs, including remote SNs. All preoperative scans were evaluated by two experienced nuclear medicine specialists (RB/BK).

Minimally invasive esophagectomy was performed according to the site’s standard of care (thoracolaparoscopic or robotic-assisted). During surgery, SNs were identified with a laparoscopic gammaprobe (Europrobe 3, PI Medical Diagnostic Equipment B.V., Raamsdonksveer, the Netherlands) and a NIR-camera (Firefly camera integrated in the da Vinci surgical system (Intuitive Surgical, Inc., Sunnyvale, California) or VISERA ELITE II Infrared Imaging System (Olympus Medical Systems, Tokyo, Japan), depending on the site where surgery was performed). The tip of the laparoscopic gammaprobe was manufactured in an angle of 30 degrees to minimize detection of background activity of the injection site.

**Fig. 1:**
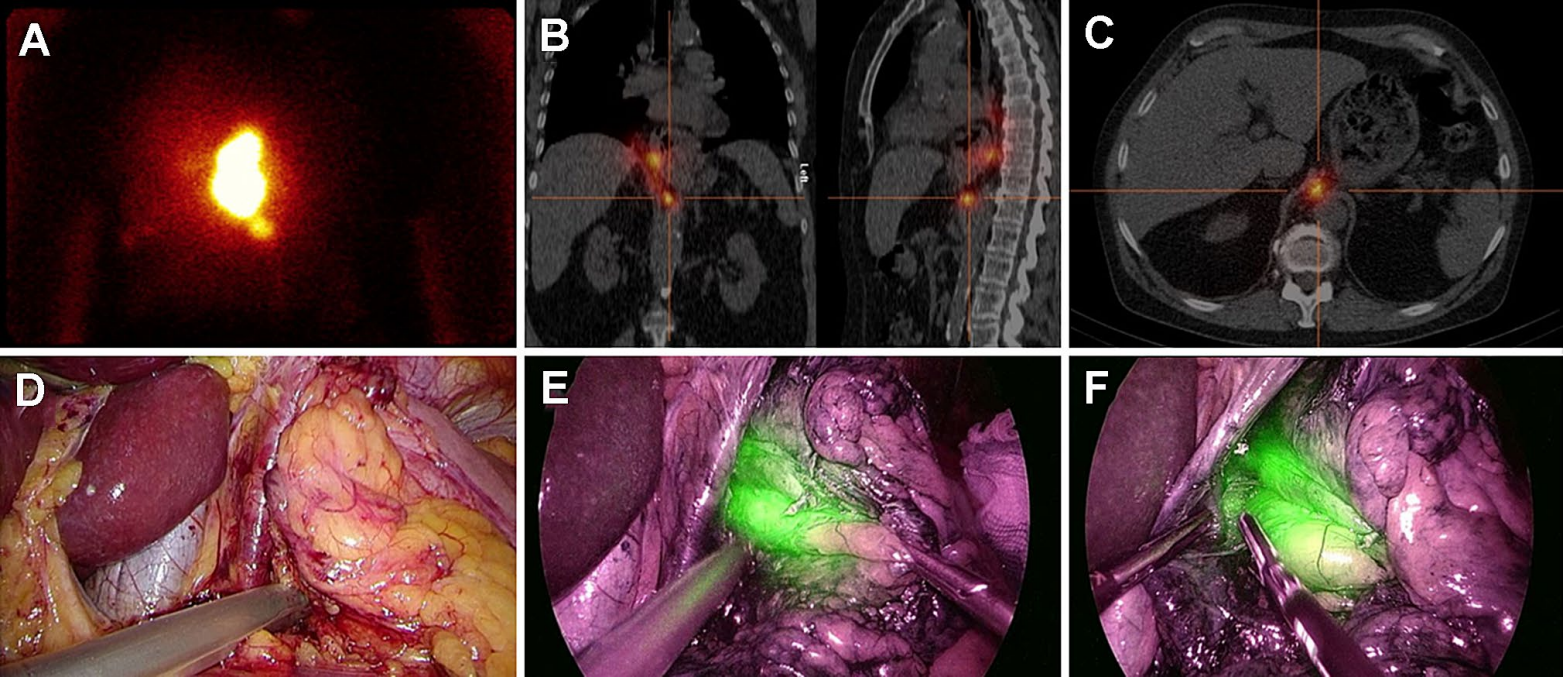
Identification of a retrocrural located sentinel node. A: Lymphoscintigraphy 120 min after injection of the hybrid tracer showed the injection site and a sentinel node located below. B + C: This was combined with a SPECT/CT of the thorax and abdomen to detect the exact sentinel node location. D: High radioactivity uptake was confirmed with the laparoscopic gammaprobe during the abdominal phase of surgery. E: The sentinel node was also clearly visualized as indocyanine green positive after switching the camera view to near-infrared. F: Laparoscopic resection of the sentinel node was started while visualized with the near-infrared camera

Esophagectomy with gastric tube reconstruction consists of a thoracic and abdominal phase (and if indicated a cervical phase), with the order depending on primary tumor localization. In this study, both phases started with SN identification and resection, followed by the standard surgical procedure. SN identification started based on preoperative imaging. After a SN was identified with the gammaprobe, camera view was switched to the NIR-camera to confirm ICG positivity and the SN was resected. Gammaprobe measurement was repeated ex-vivo for confirmation of a high radioactive uptake using a second, handheld gammaprobe. In addition to SNs detected on preoperative imaging, the peritumoral region was carefully inspected with the NIR-camera to localize SNs not visualized on preoperative imaging due to high tracer activity of the injection site. ICG positive peritumoral lymph nodes were also resected and ex-vivo radioactive uptake was measured with the gammaprobe. After SN identification and dissection was finalized, the thoracic/abdominal cavity was checked with the gammaprobe and NIR-camera to confirm absence of remaining SNs and esophagectomy with gastric tube reconstruction was completed with an intrathoracic or cervical anastomosis (Fig. 1/Online Video 1). After surgery was completed, absence of radioactivity in the esophageal resection specimen was confirmed (not taking into account radioactivity at the injection site).

Lymph node stations were classified according to the 8^th^ edition of the AJCC esophageal cancer staging system. Resected lymph node stations were: 2 on indication, 4, 7–9, 15–20. In addition, lymph nodes in the hepatoduodenal ligament and aortopulmonary window were resected. Lymph nodes stations were sent separately for histopathologic review, except for stations in close proximity to the primary tumor site, these were marked with beads/sutures to avoid damage to the circumferential resection margin.

### Histopathological evaluation

The esophageal resection specimen was processed according to the current standard of care. Additionally, all SNs were totally embedded in paraffin and cut at 3 levels, while non-SNs were cut at 1 level, and all slides were stained with hematoxylin and eosin for morphologic evaluation of metastases by dedicated pathologists (SM/LB). In case no metastases were observed, additional immunohistochemical keratin staining (AE1-3) was performed to detect the presence of micrometastases.

### Outcome parameters

Primary endpoints were: (1) percentage of patients with detectable SNs, either on preoperative imaging or detected during surgery; (2) number of SNs per patient, location documented; (3) concordance of preoperative imaging, and intraoperative fluorescence and gammaprobe-based SN detection; and (4) additional detection of SNs with the NIR-camera, not detected preoperatively on SPECT/CT or intraoperatively with the gammaprobe. Secondary endpoints were: (1) number of tumor positive (non)SNs; (2) number of resected (non)SNs; (3) additional time required for SN detection and dissection and total surgical procedure time; and (4) adverse events during 90 days of follow-up.

### Statistics

Since this was a pilot study, no formal sample size calculation was performed and a sample size of 5 patients was considered sufficient to evaluate feasibility. Statistical analyses were performed using the Statistical Software Package IBM SPSS Statistics version 25.0.0.2 for Windows (SPSS, Chicago, Illinois, USA). There were no missing data or loss to follow-up. No statistical comparisons were made; only descriptive statistics, medians with minimum and maximum values or interquartile ranges, were reported.

## Results

Five patients with histopathologically confirmed high-risk T1b EAC, with no evidence of lymph node or distant metastasis, were included between July 2018 and July 2019. Histopathological evaluation of all endoscopic resection specimens revealed at least one high-risk feature that indicated additional surgery (Table 1).

**Table 1:** Patient and tumor characteristics (N = 5)

Patient characteristics					
Male sex, n(%)	3 (60)				
Age, median(range)	56 (41–76)				
Body mass index, median(range)	27.8 (21.7–35.1)				
ASA score, n(%)					
1	1 (20)				
2	4 (80)				
3	0 (0)				
4	0 (0)				
Prague classification, median(range)					
C	0 (0–1)				
M	1 (0–5)				
Endoscopic resection technique, n(%)					
EMR	2 (40)				
ESD	3 (60)				
Adverse events during endoscopic resection, n(%)	0 (0)				

Tumor characteristics					
Patient	1	2	3	4	5
Tumor location in cm from bite block	40	35	38	40	37
Primary lesion type	0-IIa	0-IIa	0-IIc	0-Is	0-IIa
Secondary Paris component	0-IIc	0-IIc	-	-	0-IIc
Tumor length in mm	15	30	10	20	30
Tumor circumferential extent in %	25	25	20	25	25

Pathology characteristics					
Patient	1	2	3	4	5
Submucosal invasion depth	SM2	SM3	SM3	SM3	SM2
Tumor differentiation	Poor	Moderate	Poor	Moderate	Moderate
Lymphovascular invasion	No	Yes	No	No	No
Positive vertical (deep) resection margins	Yes	Yes	No	No	Yes
Remaining tumor in the surgical resection specimen	Yes, T3	No	No	No	Yes, T1sm3

**Fig. 2:**
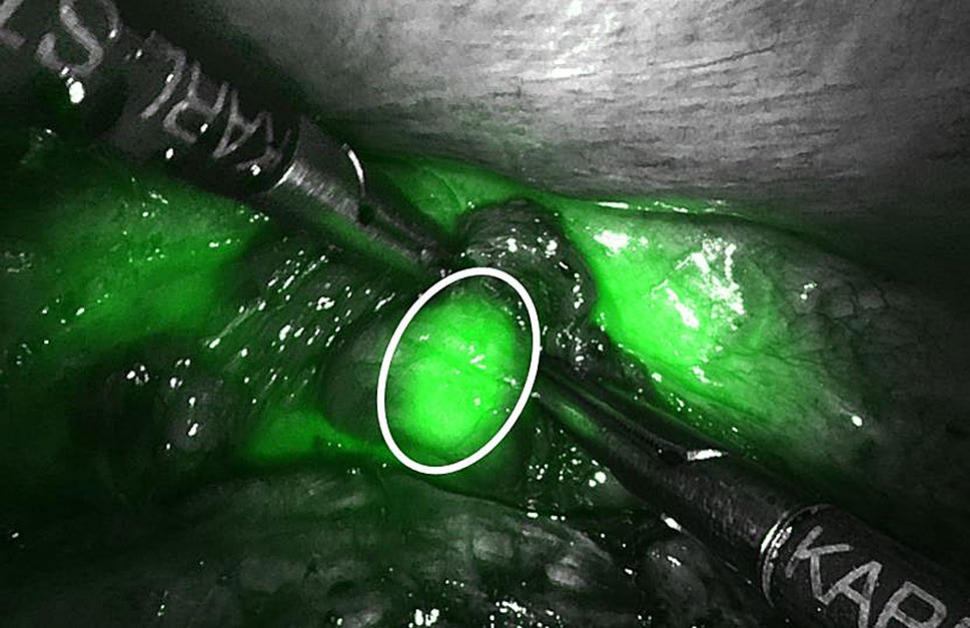
Peritumoral sentinel node detection using near-infrared light. An indocyanine green positive sentinel node (indicated by the white circle), located in distal paraesophageal station 8 near the injection site, is detected during the thoracic phase of surgery. This sentinel node was not identified preoperatively on imaging, nor was it detected intraoperatively with the gammaprobe because of the high background radioactivity around the injection site

SNNS followed by minimally invasive esophagectomy with an intrathoracic anastomosis was performed in all patients a median of 92 days (range 88–104) after the endoscopic resection. Endoscopic injection of the tracer was feasible in all patients with a median procedure time of 13 min (range 8–17). SNs were identified in all patients, both on preoperative imaging (median of 2 SNs in median 2 lymph node stations, range 1–2) and during surgery (median 3 SNs (range 2–7) in median 2 lymph node stations (range 1–4), Table 2). The median (interquartile range) in-vivo laparoscopic gammaprobe count rate was 460 (140–700) and 439 (116–649) ex-vivo after resection of the SN. The concordance between preoperative imaging and intraoperative SN detection was high: all SNs detected on preoperative imaging could be identified intraoperatively. One SN was not approached during surgery as determined in a multidisciplinary meeting (Table 2). All SNs detected intraoperatively with the gammaprobe were also identified as ICG positive with the NIR-camera (Table 2). In two patients additional SNs located near the injection site were identified with the NIR-camera (Table 2, Fig. 2). These SNs were not detected on preoperative imaging, nor were they detected during surgery with the laparoscopic gammaprobe due to the high background radioactivity of the injection site, but high radioactivity was confirmed ex vivo. After surgery was completed, absence of in vivo radio- and fluorescence-activity was confirmed, as was absence of radioactivity in the esophageal resection specimen (not taking radioactivity at the injection site into account). Total median surgery procedure time was 6.9 h (range 6.2–8.5) including median 46 min (range 31–68) required for SNNS. No acute adverse events occurred during the additional SNNS procedures or during esophagectomy and reconstruction.

**Table 2:** Concordance of SN detection: imaging-, probe- and indocyanine-green-based detection

SN stations	Patient 1	Patient 2	Patient 3	Patient 4	Patient 5
1—supraclavicular				Imaging*	
2R—right high paratracheal				Imaging, probe & ICG(1 SN)	
5—aortopulmonary		Imaging, probe & ICG(1 SN)			
7—subcarinal					Imaging, probe & ICG(2 SN)
8 M—mid paraesophageal					ICG(1 SN)**
8L—low paraesophageal		Imaging, probe & ICG(4 SN)			
15—diaphragmatic	Imaging, probe & ICG(2 SN)				
16—paracardial	Imaging, probe & ICG(1 SN)			ICG(5 SN)**	
18—common hepatic				ICG(1 SN)**	
20—celiac trunk			Imaging, probe & ICG(2 SN)		
Total number of resected SNs	3	5	2	7	3

*This SN was located outside of the surgical resection area and was therefore not resected

**These SNs were detected with ICG only, because of their proximity to the injection site with its high background radioactivity

During histopathological evaluation, in total median 49 lymph nodes (range 44–66) were identified per patient. Median 5 lymph nodes (range 3–19) were identified per patient in the resected SN stations. None of the resected SNs showed signs of (micro) metastases and none of the other lymph nodes showed tumor positive cells. In 2 of the 3 patients with a known incomplete endoscopic resection, remaining tumor was found in the esophageal resection specimen (1: T3, poor differentiation, no lymphovascular invasion; 2: T1sm3, moderate differentiation, no lymphovascular invasion). The surgical resection margins were free of tumor in both patients.

Patients were hospitalized for a median of 13 days (range 10–50). All patients experienced adverse events during 90 days of follow-up and one patient had a serious adverse event (Supplementary material 1). All patients were alive after 90 days of follow-up.

## Discussion

This study investigates SNNS with a hybrid tracer of technetium-99 m and indocyanine green combined with nanocolloid in high-risk T1b EAC patients and shows that SNNS is feasible and safe. Moreover, the addition of ICG to the ^99m^Tc-nanocolloid tracer seems to improve SN detection by enhancing visualization of SNs located near the injection site, and by visual affirmation of probe detected remote SNs and completeness of SN resection during surgery. Since ICG can only be visualized intraoperatively and in close proximity to the fluorescence-active nodes, the radioactive tracer remains important for preoperative detection of remote SNs and intraoperative guidance to the regions of interest.

SNNS for esophageal cancer faces several challenges. The lymphatic network of the esophagus is multidirectional with a wide variation in SN locations [10]. Additionally, an esophagectomy is a full day surgical procedure consisting of separate abdominal and thoracic phases, which requires long-lasting durability of the tracer. Furthermore, to cover all lymphatic pathways, upper endoscopy is required for precise injection of the tracer in four quadrants around the endoscopic resection scar. Lastly, logistics and planning can be challenging, because multiple procedures have to be planned in a short timeframe before surgery.

The number of studies on SNNS in esophageal cancer is limited, and this holds even more for studies on SNNS preceded by endoscopic resection of the tumor^12^. Two meta-analyses, including different histologic subtypes and multiple clinical stages, report high detection and accuracy rates (> 80%) [18, 19]. Regarding clinical stage, failure of SN mapping is reported more frequently in clinically more advanced tumors. Therefore, T1 tumors are considered the most suitable candidates for SNNS [10]. Four studies specifically focusing on T1 tumors (EAC/ESCC), showed promising results with detection rates of 93–100%, a sensitivity of 69–92% and an accuracy of 80–97% [12, 13, 20, 21].

This study is the first on SNNS in high-risk T1b EAC with a *hybrid* tracer of ^99m^Tc and ICG combined with nanocolloid. SNNS with this hybrid tracer has shown promising results in other malignancies [14, 15]. Separate injections for the radioactive tracer combined with nanocolloid on the day before surgery and for ICG alone during surgery have also been used, for instance in early stage gastric cancer [16]. However, ICG alone has a short durability (up to 3.5 h) [22]. Given the long duration of esophageal surgery consisting of two separate phases both starting with SN detection, the use of ICG alone would require two additional upper endoscopies during surgery, one at the start of each phase, to ensure consistent visibility of ICG.

In the current study, there were no patients with tumor positive lymph nodes. When combining the results of the present study with preceding SNNS studies of our research group, 2 out of 12 patients (17%) with a high-risk T1b EAC had LNM [12]. This percentage is in line with two retrospective series by our group on LNM risk in high-risk T1b EAC [2, 5]. In these studies, histopathological evaluation of the endoscopic resection specimen was performed very precisely, since this determined the need for additional treatment.

Strengths of this study are its multicenter setting with involvement of a consistent multidisciplinary, experienced research staff. In addition, all study procedures were attended by one research fellow to ascertain uniformity throughout all participating centers. Moreover, the laparoscopic gammaprobe was customized with a 30 degree angle on the tip, and using a combined tracer precision of injection and durability of the tracer were assured. Lastly, by including patients diagnosed with high-risk T1b EAC based on histopathological evaluation of an endoscopic resection specimen, we adhered to current clinical practice.

Some limitations should be addressed. Due to the absence of tumor positive SNs, we have no confirmation that we identified the correct lymph nodes as SNs. Even so, the absence of tumor positive lymph nodes confirms the need for tailored treatment for this patient category. Secondly, in one patient that had an incomplete endoscopic resection, final pathology of the surgical resection specimen showed a T3 EAC. Since in this study all patients would be planned for esophagectomy regardless of the SNNS result, an incomplete endoscopic resection was not considered an exclusion criterion. For future studies with additional treatment depending on the SNNS result, only patients with a radical endoscopic resection of T1b EAC will be considered for inclusion. Moreover, patients were not consented for additional invasive procedures, such as separate excision of SNs located outside the standard surgical resection area. Therefore, in the current study, one SN was not approached. Since this supraclavicular SN was located just distally to a tumor negative SN at station 2R, it was decided to closely monitor this lymph node during follow-up and not schedule a separate excision. Lastly, for this pilot feasibility study a small sample size of five patients was considered sufficient. Proper validation of the SNNS technique would be difficult. Such a study would require long-term follow-up in a high number of patients, since only a small number of patients with high-risk T1b EAC will have LNM.

**Fig. 3:**
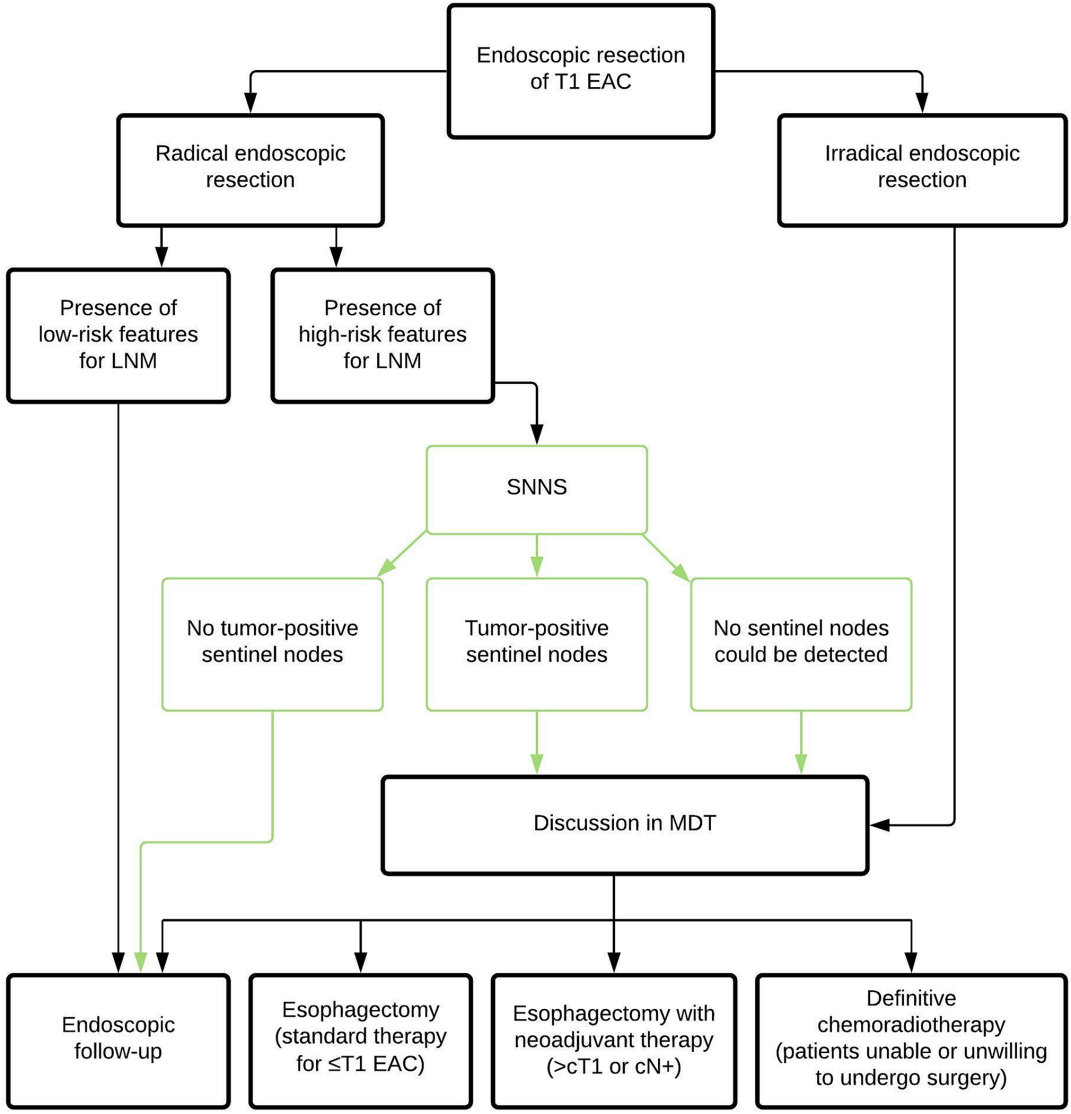
Treatment flowchart for patients with T1 esophageal adenocarcinoma including the potential role of sentinel node navigation surgery

Future research should therefore focus on applying this new treatment algorithm (radical endoscopic resection with additional treatment based on the SNNS result) in a prospective series of high-risk T1b EAC patients (Fig. 3). In the majority of patients no tumor positive sentinel nodes will be identified and esophagectomy will thus be omitted. Given the difficulty of proper validation of SNNS, these patients will be monitored closely with stringent endoscopic follow-up including endoscopic ultrasound examination to monitor the occurrence of locoregional recurrence and LNM, as well as the function and motility of the esophagus and quality of life of patients (Netherlands Trial Register; NL8100). In parallel, SNNS will be evaluated in the exceptional patient category of high-risk T1b EAC *with* clinical suspicion of LNM (clinically staged as T1N1) (Netherlands Trial Register; NL8558). Currently, a wait-and-see policy with only endoscopic follow-up after radical endoscopic resection is also being evaluated (clinicaltrials.gov; NCT03222635). Ultimately, one would want to compare the new treatment algorithm with SNNS to the wait-and-see policy with endoscopic follow-up only in a clinical impact study to evaluate beneficial effects on disease-free and overall survival.

In conclusion, SNNS with technetium-99 m-ICG-nanocolloid seems feasible and safe in patients with high-risk T1b EAC, and might be a suitable strategy to tailor additional treatment after radical endoscopic resection has been performed. ICG seems to be of additive value for localizing peritumoral SNs. Whether this approach can optimize selection for esophagectomy needs to be studied in future research.

## Supplementary Information

Below is the link to the electronic supplementary material.

Supplementary file1 (DOCX 16 KB)

Supplementary file2 (MP4 110154 KB)
