# Supplementary material for: Feasibility of sentinel node navigated surgery in high-risk T1b esophageal adenocarcinoma patients using a hybrid tracer of technetium-99 m and indocyanine green
Source: Surg Endosc. 2021 May 27;36(4):2671–9. doi: 10.1007/s00464-021-08551-6 (PMC8921120; doi:10.1007/s00464-021-08551-6)
Supplement: Supplementary file 1 — Supplementary file1 (DOCX 16 KB) [file 464_2021_8551_MOESM1_ESM.docx]

**Supplementary material 1** (Serious) adverse event description and subsequent interventions

| Patient 1: | - Paroxysmal atrial fibrillation: start edoxaban. |
| --- | --- |
| Patient 2: | - Atrial fibrillation and (sub) segmental pulmonary embolism: start edoxaban. - Anastomotic stricture: multiple endoscopic dilations (>5) |
| Patient 3: | - Serious adverse event: Prolonged hospitalization because of multiple adverse events after surgery: (1) chylous leakage: second surgery to clip the thoracic duct; (2) anastomotic leakage connected with a necrotic cavity: endoscopic drainage and flushing; (3) anastomotic stricture: endoscopic dilation. |
| Patient 4: | - Anastomotic stricture: multiple dilations (>5) and stent placement |
| Patient 5: | - Bacteremia with enterobacter aerogenes: antibiotic therapy |
